# Solitary fibrous tumor of the liver: a case report

**DOI:** 10.1186/1477-7819-9-37

**Published:** 2011-03-29

**Authors:** Ke Sun, Jian-Ju Lu, Xiao-Dong Teng, Li-Xiong Ying, Jian-Feng Wei

**Affiliations:** 1 Department of Pathology, the First Affiliated Hospital, College of Medicine, Zhejiang University, 79 Qingchun Road, Hangzhou, Zhejiang, 310003, PR China; 2 Department of Hepatobiliary Surgery, the First Affiliated Hospital, College of Medicine, Zhejiang University, 79 Qingchun Road, Hangzhou, Zhejiang, 310003, PR China

## Abstract

Hepatic solitary fibrous tumor (SFT) is a rare tumor originating from the mesenchyme. Here we report a new case of SFT in the liver and review the clinical presentation, radiological and operative findings, diagnosis, treatment, and outcome. The patient was a 59-year-old man who presented with progressive fatigue for 3 months and an abdominal mass for 3 days. On laboratory tests, no abnormality was detected except that abdominal ultrasonography revealed a 9.0 × 6.2 cm hypoechogenic mass in the left lobe of the liver. A computed tomographic scan confirmed a hypodense lesion in the left lobe of the liver. The patient underwent left hepatectomy. SFT was diagnosed on the basis of histopathological findings. The patient was free from all symptoms and had no signs of local recurrence after 24 months' follow up.

## Background

Solitary fibrous tumor (SFT) is a rare spindle-cell neoplasm of mesenchymal origin, first described by Klemperer and Robin in the visceral pleura in 1931 [1]. It usually is found in the thoracic cavity and pleura, but, rarely, it can involve other organs such as the mediastinum [2], the skin [3], soft tissue [4], the thyroid gland [5], the orbit [6], and others. Although most solitary fibrous tumors have benign behavior, some may have malignant features such as metastasis and recurrence. Clinical or radiological findings are not specific and cannot exclude malignancy. Preoperative cytology may be inconclusive or misleading. Currently, the prevailing view is that immunohistology, including CD34 and vimentin, should be used to precisely diagnose SFT [7]. The outcome of an SFT of the liver is mostly related to resectability [8]. Thus, complete surgical removal of the neoplasm is most commonly proposed. We report a case of SFT of the liver and review the literature to date.

## Case Presentation

**Figure 1:**
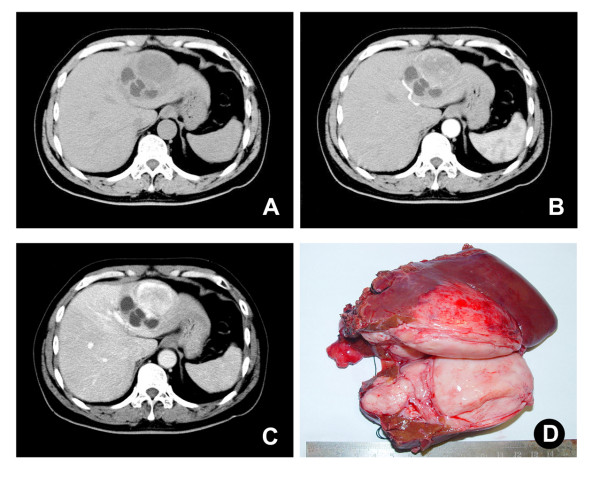
**Single mass in the left hepatic lobe**. (A) CT scan demonstrated a mass (9.0 × 6.2cm) in left hepatic lobe. (B) and (C) Contrast enhancement in arterial and portal phases was found in the mass. (D) Grossly, a large, gray-white, lobulated, well-circumscribed, partially encapsulated mass was removed after hepatoectomy.

A 59-year-old man was admitted to our hospital because of progressive fatigue for 3 months and an abdominal mass for 3 days. The patient had no history of viral hepatitis. Laboratory tests, including routine biochemistry, liver function, and tumor markers, were normal. A plain chest X-ray was normal. Abdominal ultrasonography revealed a 9.0 × 6.2 cm hypoechogenic mass in the left lobe of the liver. Computed tomography (CT) demonstrated a large heterogeneous circumscribed mass in the left hepatic lobe and contrast enhancement in the arterial and portal phases (Figure 1A, B, C). Left hepatectomy was performed, and the patient recovered without complications.

**Figure 2:**
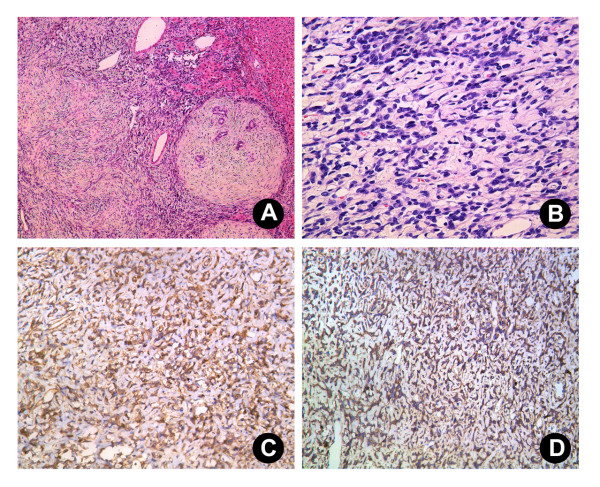
**Histological section of the SFT**. (A) and (B) The tumor was composed principally of spindle cells arranged in short, ill-defined fascicles in some zones and randomly in others (A; H & E 10×; B; 40×). Immunohistochemically, the tumor cells were strongly positive for CD34 (C; 20×) and CD99 (D; 20×).

Grossly, the tumor was a large, gray-white, lobulated, well-circumscribed partially encapsulated mass, measuring 9 × 7 × 6 cm. In the peripheral liver parenchyma, the vascular structure and bile duct were compressed, and no cirrhosis or fibrosis was observed (Figure 1C). Histologically, the tumor was composed principally of spindle cells arranged in short, ill-defined fascicles in some zones and randomly in others (Figure 2A, B). They were intermingled with striking areas of hyalinization. The vascular pattern varied from narrow vascular clefts to gaping, branching vascular channels. Cystic degeneration was present. Little mitotic activity was observed (fewer than 1-2 mitoses in 10 high-power fields (HPF)), and these foci showed more dense cellularity and more nuclear atypia; this was considered to represent low-grade malignant transformation. This tumor also showed irregular infiltration of the peripheral liver (Figure 2A). Little inflammatory cell infiltration without necrosis was seen.

Immunohistochemically, the tumor cells were strongly positive for CD34 (Figure 2C), CD99 (Figure 2D), Bcl-2, and vimentin and negative for smooth muscle actin (SMA), CD31, cytokeratin, S-100, CD117, and epithelial membrane antigen (EMA). A solitary fibrous tumor of the liver was diagnosed pathologically. The patient was free from any symptoms and without local recurrence for 24 months.

## Discussion

Primary solitary fibrous tumor of the liver is an extremely rare neoplasm. Individual case reports are infrequent. The first description of this type of tumor involving the liver may be that provided by Nevius and Friedman in 1959 [9]. A review of the literature revealed fewer than 40 reported SFTs of the liver to date [7,8,10]. These tumors mainly occurred in women, at a ratio of 71.7 percent female to 28.3 percent male. The mean age was 58.9 years old. The tumor can be found in either the right or the left hepatic lobe. Clinically, most patients may be asymptomatic, whereas other patients are symptomatic with abdominal pain, abdominal fullness and mass, weight loss, and fatigue when the tumor grows. Some present with alterations of liver tests and compression of biliary ducts leading to cholestasis [11]. In general, the clinical presentation of patients with SFT usually is mild and not distinctive from that of other lesions of the liver.

The radiological findings may suggest the diagnosis of SFT, but benign or malignant hepatic tumors such as hepatocellular carcinoma, sarcoma, leiomyoma, and inflammatory pseudotumor may have similar features [11]. Thus, the radiological findings are nonspecific and cannot distinguish between benign and malignant tumors. Therefore, in the present case, the diagnosis of SFT of the liver was based on the association of typical histological and immunohistochemical features. CD34 positivity distinguishes SFT from other spindle-cell tumors [12], and it is necessary to combine other markers for differential diagnosis. In our case, the tumor cells were strongly positive for CD34, CD99, Bcl-2, and vimentin and negative for smooth muscle actin (SMA), CD31, cytokeratin, S-100, CD117, and epithelial membrane antigen (EMA).

The pathologic features of the SFT of the liver described here resemble those described for solitary fibrous tumors of other locations [13]. Typical SFTs show a patternless architecture characterized by a combination of alternating hypocellular and hypercellular areas separated from each other by thick bands of hyalinized, somewhat keloidal collagen and branching haemangiopericytoma-like vessels. These features differentiate SFTs from other liver mesenchymal tumors. Immunohistochemically, SFTs consistently express CD34, are variably positive for CD99 and Bcl-2, and lack cytokeratin or other mesothelial markers. The presence of mitotic figures is associated with but not predictive of aggressive clinical behavior [14]. The histologic features of this malignancy include high cellularity and mitotic activity, pleomorphism, necrosis, and local invasion [8].

The differential diagnosis includes leiomyoma (consists of intersecting bundles of smooth muscle cells; SMA positive, CD34 negative) [15], inflammatory pseudotumor (consists of myofibroblasts, fibroblast cells mixed with inflammatory cells, predominantly plasma cells, lymphocytes, as well as eosinophils; SMA positive, vimentin positive, and CD34 negative) [16], fibrosarcoma (forms a "herringbone" pattern; CD34 negative), and stromal tumor (CD117 and CD34 positive) [11].

Although most cases have benign clinical behavior, there is no strict correlation between histological findings and biological behavior. Some may have malignant histological features and recur locally or metastasize [17]. Therefore, a complete surgical resection is the first choice for treatment and is curative in most cases. Follow-up surveillance is necessary. According to the limited literature on hepatic SFTs, all but three patients underwent surgical resection. There is only one report of distant metastasis to the bone, and it was successfully treated with chemotherapy [17]. Among 92 cases of extrathoracic SFT, ten cases with atypical features including high cellularity, >4 mitoses/10 HPF, nuclear pleomorphism, and necrosis were associated with aggressive clinical behavior. Among these ten patients, eight had local recurrence or distant metastases [14]. Interestingly, one widely metastatic tumor did not have any atypical features in the primary lesion but acquired four such features in the metastatic foci. These findings confirm that the behavior of extrathoracic SFTs is unpredictable. Due to the rarity of this tumor, the prognosis has not been well defined [8]. Therefore, patients with SFTs in any location require careful long-term follow up, and it is probably unwise to regard any such lesion as definitely benign [14].

## Conclusion

We observed a rare case of SFT of the liver. Correct interpretation of unique pathological findings and CD34 immunoreactivity plays a significant role in differentiating SFT from other spindle-cell neoplasms of the liver. A complete surgical resection is the treatment of choice and is curative in most cases, and follow-up surveillance is necessary. The outcome of SFTs is mostly related to resectability rather than pathologic grade or tumor size. Given the limited number of cases reported in the literature, it is still difficult to establish the long-term prognosis of this disease.

## Competing interests

The authors declare that they have no competing interests.

## Authors' contributions

KS, JFW, and XDT conceived the concept, participated in drafting the manuscript, and conducted critical review. JJL took part in the care of the patient, assembled data, and participated in writing the manuscript. LXY carried out the histopathological evaluation and reviewed pathology. All authors read and approved the final manuscript.

## Consent

Written informed consent was obtained from the patient for publication of this case report and any accompanying images. A copy of the written consent is available for review by the Editor in Chief of this journal.
